# Why health apps fail: the role of smartphone proficiency in mHealth resistance

**DOI:** 10.3389/fpubh.2026.1829773

**Published:** 2026-05-26

**Authors:** Satoshi Inagaki, Kenji Kato, Hisafumi Yasuda

**Affiliations:** 1 School of Data Science, Nagoya City University, Nagoya, Aichi, Japan; 2 Kobe University Graduate School of Health Sciences, Kobe, Hyogo, Japan; 3 Faculty of Nursing, Kobe Women’s University, Kobe, Hyogo, Japan

**Keywords:** digital divide, digital literacy, eHealth, health apps, psychological resistance, smartphone, technology acceptance

## Abstract

**Background:**

Mobile health apps offer significant potential for health promotion, yet initial adoption remains limited. While prior research has identified app-specific barriers, the perceived usefulness (PU) and psychological resistance toward health apps compared to everyday commercial apps, as well as the foundational role of users’ smartphone proficiency, remain understudied.

**Objective:**

This study aimed to compare users’ perceptions of health apps with those of other app categories and to examine whether smartphone proficiency or health-related status was consistently associated with PU and psychological resistance.

**Methods:**

A cross-sectional internet survey was conducted among 717 adults in Japan. We compared the PU of health apps with news, video streaming, social media, and gaming apps. Furthermore, we compared psychological resistance toward a physician-recommended health app with a discount-incentivized shopping app. Ordinal logistic regression was used to identify independent predictors of PU and resistance.

**Results:**

Health apps were perceived as moderately useful (mean PU = 6.2), similar to social media (adjusted *p* = 0.345) but inferior to news apps (adjusted *p* < 0.001). Psychological resistance toward a physician-recommended health app (mean = 2.69) was virtually no different to resistance toward a discount-incentivized shopping app (mean = 2.66, *p* = 0.521). Practical barriers, such as device storage (43.1% vs. 48.3%) and privacy concerns (42.3% vs. 46.0%), were the primary barriers for both, rather than a lack of perceived necessity (23.2% vs. 31.2%). Health-related indicators showed only limited associations with app acceptance. In contrast, higher smartphone proficiency independently predicted both increased PU [adjusted odds ratio (aOR) = 4.69, 95% CI: 1.90–11.52, *p* < 0.01] and diminished psychological resistance (aOR = 0.12, 95% CI: 0.05–0.31, *p* < 0.001).

**Conclusion:**

Health apps face practical adoption barriers similar to those of everyday commercial apps. Initial acceptance appears to be more consistently shaped by foundational digital proficiency than by health-related status. To support equitable digital health interventions, public health strategies should prioritize strengthening basic digital competencies in the population.

## 1 Introduction

Mobile health (mHealth) apps are widely recognized as powerful tools that empower individuals to proactively manage their health, demonstrating utility in areas such as weight management and exercise promotion (1–3). While some reports indicate that approximately 40–50% of adults use them in some capacity (4–6), the widespread adoption and sustained use of mHealth apps face persistent challenges. Many users abandon these apps shortly after initiation (3, 7), and a substantial portion of the population has never adopted them at all. This highlights a critical need to understand the factors hindering initial adoption.

Research on mHealth adoption often uses technology acceptance models (8, 9), which suggest that perceived usefulness (PU)—the degree to which a person believes that using a particular system would enhance their performance—and perceived ease of use are important factors in technology acceptance (10–13). However, to understand why initial adoption fails, it is equally important to examine user resistance. Resistance goes beyond a passive refusal to adopt; it encompasses active opposition or rejection arising from a perceived incongruence between the user’s goals and the technology (14). Previous studies have characterized resistance to health apps through various barriers, including data privacy concerns (15–17), the burden of manual data entry (18, 19), and app complexity (19, 20). However, little research has evaluated health apps in relative terms against other application types, so the practical weight of these barriers remains unclear. Crucially, the perceived weight of these barriers may depend on when the app provides rewards. Entertainment and social media apps offer immediate gratification and direct emotional benefits (21). In contrast, the benefits of health apps typically accumulate over time (22, 23), requiring sustained effort and behavioral change (18, 22). The difference in gratification timing is crucial for understanding why consumers may experience psychological resistance or lower intention to adopt health apps, even if they recognize their usefulness.

Furthermore, while traditional adoption models often provide a robust framework for evaluating app-specific perceived ease of use (10), they inherently assume a baseline of smartphone operation skills. Without these basic skills, users may find it difficult to judge the usability of an app. Therefore, beyond the specific app’s perceived usability or health-related status (24), general smartphone proficiency may act as a critical antecedent that shapes users’ perceptions of ease of use and, consequently, their willingness to adopt the apps.

Like many high-income nations, Japan has high overall digital connectivity, but digital access and use remain strongly age-stratified (25, 26). Given Japan’s rapidly aging population and the high number of people who feel insecure about their digital skills, as shown in international surveys (27), Japan is an important stage for examining how the universal digital gap impacts health equity and digital inclusion.

Building on these perspectives, this study aims to provide a comprehensive understanding of how health apps are perceived and why resistance to adoption occurs. Specifically, we address the following research questions:

1. How does the PU of health apps compare with that of other types of applications, such as news, social media, and gaming apps?
2. How does psychological resistance toward a physician-recommended health app differ from resistance toward a commercial app offering discount incentives?
3. To what extent is smartphone proficiency, relative to health-related status, associated with the PU of and psychological resistance to health apps?

By focusing not only on app-specific factors but also on users’ foundational digital competencies, this study seeks to expand the understanding of mHealth technology acceptance. The findings will inform the development of more effective adoption strategies and equitable digital health policies.

## 2 Materials and methods

### 2.1 Research design

A cross-sectional internet-based survey was conducted among those preregistered with a research firm. The survey protocol was developed in accordance with the ‘Checklist for Reporting Results of Internet E-Surveys (28).

### 2.2 Participants and recruitment

Participants were recruited through a research firm with an established record of academic collaboration and an extensive panel of respondents. Individuals voluntarily register as monitors to receive modest compensation for participating in research studies, marketing research, and product development.

Eligibility criteria included being aged 18 years or older, residing in Japan, being able to read Japanese, and owning a smartphone. No specific exclusion criteria were applied. To ensure representativeness, a quota sampling approach was employed to match the age and gender distribution of the Japanese population, based on the 2022 estimates published by the Statistics Bureau of Japan (29).

A two-step recruitment process sampling design was implemented. All sampling, recruitment, and survey procedures were conducted entirely within the web platform operated by the research firm. In the first step, individuals registered with the firm were shown a survey invitation titled “Academic Survey on Subjective Impressions and Attitudes,” which was displayed alongside other available survey opportunities on the firm’s platform. Those who voluntarily clicked on this invitation were directed to a screening questionnaire that included items on age, gender, and smartphone ownership. This process enabled identification of respondents who met both the eligibility criteria and the target quotas. In the second step, individuals who qualified were presented with a detailed consent form describing the study’s purpose, procedures, estimated time commitment (approximately 15 min), data protection and anonymity assurances, and compensation details. Only individuals who explicitly agreed to participate proceeded to the main questionnaire. To ensure data quality, responses were included in the final sample only if they met quality control criteria applied by the research firm, which included checks for excessively rapid completion, invariant or straight-line responding, and failed attention check questions.

### 2.3 Data collection procedure

The survey was conducted via participants’ personal devices after logging into the research firm’s platform. Unique user IDs and platform safeguards prevented individuals from participating more than once. Participants received compensation equivalent to less than $1.00 USD upon survey completion, in accordance with the research firm’s regulations.

The questionnaire was pretested to confirm the clarity of items and to estimate the time required for completion. To promote accurate responses, the survey was designed to include multiple embedded attention check items that screened for attentiveness (e.g., simple arithmetic problems or specific response instructions).

Once the target sample size was reached, the research firm screened the responses for quality—removing entries based on the criteria detailed previously—and then closed the survey and provided the cleaned dataset to the researchers in Microsoft Excel format.

### 2.4 Measures

Most survey items were developed by the authors.

Participants were asked about their smartphone usage, including total years of use, average daily usage time, self-rated proficiency, and perceived difficulty. Self-rated smartphone proficiency, the primary independent variable, was measured using a single item:

*“How would you rate your overall smartphone skills?”*

Participants responded on a four-point scale: (1) Not at all proficient, (2) Insufficiently proficient, (3) Moderately proficient, and (4) Highly proficient.

They were also asked about their experience with five categories of smartphone applications: health, social networking, gaming, news, and video streaming.

The Perceived Usefulness of several key app categories was assessed on a 10-point Numerical Rating Scale (NRS) from 1 (“strongly disagree”) to 10 (“strongly agree”). To enable direct comparison, participants rated five app types—Video Streaming, Gaming, Social Networking, News, and Health apps — on the core item:

*“I feel this type of app is useful to me.”*

In addition to PU, participants also provided exploratory ratings for other affective impressions (interest, attractiveness, fun, and obsession) using the same NRS format. For apps a participant had never used, these ratings were collected as “expected” impressions (e.g., expected fun). These additional data are reported in Supplementary files.

Participants’ Psychological Resistance was operationalized as an overall reluctance to an app installation recommendation. This was measured in two different scenarios. First, for a health-specific context, they were asked:

*“If a physician recommended that you install a health app for your health management, to what extent would you feel resistant to installing it?”*

Second, to provide a commercial context for comparison, they were asked a similar question regarding a shopping app:

*“If you were introduced to a new app that offers discounts when shopping or dining, to what extent would you feel resistant to installing it?”*

For both scenarios, responses were recorded on a 5-point NRS, anchored from 1 (“no resistance at all”) to 5 (“very strong resistance”). Additionally, to explore the specific cognitions underlying this overall resistance score, participants were subsequently asked to select their reasons for this resistance from a predefined list of 12 items.

These single-item measures were intentionally chosen to minimize respondent burden and prevent survey fatigue. While noting their psychometric constraints, recent methodological literature suggests that single-item measures can offer acceptable validity in specific research contexts (30). Therefore, this approach was considered appropriate for capturing broad practical trends, aligning with the exploratory nature of the current study.

Additional items included sociodemographic variables (e.g., age, gender, education level, occupational status, regular hospital visit, and self-rated economic status). Physical activity habits were assessed based on self-reported frequency. Self-rated health was measured on a 100-point scale. Psychological wellbeing was measured with the Japanese version of the WHO-5 Wellbeing Index (score range: 0–25), a validated five-item scale (31).

Finally, participants’ experiences with health apps were assessed. This included their usage status (current, past, or never used), and for current users, data on their frequency of use, duration of use, and tracked features. Reasons for discontinuation were also collected from past users.

### 2.5 Statistical analysis

Descriptive statistics were used to summarize participant characteristics, PU of various app categories, and smartphone usage patterns.

Differences in PU across the five app categories were analyzed using a Friedman test, followed by *post-hoc* pairwise Wilcoxon signed-rank tests with Bonferroni correction. To compare the psychological resistance scores—measured on a 5-point ordinal scale—between the physician-recommended health app and the discount-incentivized shopping app, a Wilcoxon signed-rank test was conducted. Additionally, McNemar’s test was utilized to compare the paired binary responses regarding the specific reasons for hesitation in installing these two types of applications.

To make descriptive comparisons between groups, we conducted subgroup analyses based on age, sex, educational background, self-rated economic status, and regular hospital visits. For each subgroup, we summarized the overall mean PU and resistance scores, as well as the mean scores stratified by smartphone proficiency. We also tested interaction terms between smartphone proficiency and each subgroup variable in separate ordinal logistic regression models.

Finally, to identify the independent predictors of PU (a 10-point scale) and psychological resistance (a 5-point scale) toward health apps, we performed ordinal logistic regression analyses. The models included sociodemographic variables (age, sex, educational background, occupational status, and self-rated economic status), self-reported health indices (regular hospital visits, self-rated health status, WHO-5 wellbeing index), lifestyle factors (exercise habit), and self-rated smartphone proficiency as independent variables. We adopted ordinal logistic regression as our primary analytical method because it preserves the ordinal nature of the dependent variable, offering advantages in terms of statistical efficiency and interpretability. We evaluated the proportional odds assumption using the Brant test and found it to be violated (*p* < 0.05). However, switching to multinomial logistic regression would result in the loss of ordinal information, an increase in the number of parameters, and greater interpretational complexity (32). Given these circumstances, we opted for standard ordinal logistic regression, supported by literature suggesting that it offers a dependable summary of the average effect (32, 33).

All statistical tests were two-sided, and a *p* < 0.05 was considered statistically significant. All statistical analyses were conducted using R software (version 4.4.2).

### 2.6 Ethical considerations

This study was approved by the Ethics Committee of the Faculty of Health Sciences, Kobe University (Approval No. 1228; December 20, 2023). All procedures were conducted in accordance with the Ethical Guidelines for Medical and Health Research Involving Human Subjects issued by Japan’s Ministry of Education, Culture, Sports, Science and Technology and Ministry of Health, Labor and Welfare, as well as the Declaration of Helsinki. Written informed consent was obtained from all participants prior to their participation.

## 3 Results

### 3.1 Participant characteristics and app engagement

A total of 717 valid responses were included in the descriptive analyses. Thirty-seven participants had missing data on self-rated economic status; analyses involving this variable were therefore conducted using complete cases. No imputation was performed. Table 1 presents the demographic characteristics, health and lifestyle factors, and baseline smartphone usage patterns of the participants. The mean age of participants was 47.8 years (SD = 17.5). The distribution of age groups was as follows: 18–39 years (35.4%), 40–59 years (34.3%), and 60 years or older (30.3%), with a roughly equal gender distribution.

**Table 1:** Participant demographics (*N* = 717).

Characteristic	*N* = 717^1^
Age (years)	47.8 (17.5)
Sex	
Male	348 (49%)
Female	369 (51%)
Educational background	
High school or below	237 (33%)
College/diploma	125 (17%)
University degree	355 (50%)
Occupational status	
Employed	205 (29%)
Self-employed	63 (8.8%)
Part-time/student	173 (24%)
Unemployed/homemaker	276 (38%)
Economic status	
Very tight	149 (22%)
Tight	272 (40%)
Somewhat comfortable	212 (31%)
Comfortable	47 (6.9%)
Missing	37
Regular hospital visits	
No visits	372 (52%)
Regular visits	345 (48%)
Exercise habit	
Almost none	325 (45%)
Irregularly (1–2 days/month)	80 (11%)
Regularly (1–2 days/week)	130 (18%)
Regularly (≥3 days/week)	182 (25%)
Self-rated health status (0–100)	66.4 (21.8)
WHO-5 wellbeing index (0–25)	12.7 (6.0)
Smartphone usage duration	
<3 years	52 (7.3%)
3–<5 years	97 (14%)
5–<10 years	214 (30%)
10 + years	354 (49%)
Daily smartphone usage time	
<1 h	86 (12%)
1–2 h	115 (16%)
2–3 h	102 (14%)
3–4 h	149 (21%)
4–6 h	126 (18%)
6 + hours	139 (19%)
Smartphone proficiency	
Not at all	25 (3.5%)
Insufficiently proficient	199 (27.8%)
Moderately proficient	379 (52.9%)
Highly proficient	114 (15.9%)
Difficulty using smartphone	
Very difficult	20 (2.8%)
Difficult	157 (22%)
Not difficult	376 (52%)
Not difficult at all	164 (23%)
Health app usage status	
Never used	320 (45%)
Used to use	118 (16%)
Currently use	279 (39%)

^1^Mean (SD); n (%).

Regarding self-rated smartphone proficiency, while over half of the participants (53%) considered themselves moderately proficient, a substantial portion reported insufficient proficiency (28%) or no proficiency at all (3.5%). Smartphone proficiency also differed markedly by age group: low proficiency (Not at all or Insufficient) was reported by 12.2% of participants aged 18–39 years, compared with 37.8% of those aged 40–59 years and 46.0% of those aged 60 years or older. Furthermore, overall engagement with mHealth tools was limited, only 38.9% of participants reported currently using at least one health-related app, whereas 44.6% had never used such an app. Further details, including age-group comparisons for smartphone and app usage (Supplementary Tables 1, 2, 4), as well as specific analyses on app features and reasons for discontinuation (Supplementary Tables 3, 5, 6), are provided in Supplementary material.

### 3.2 General attitudes toward health apps

To contextualize general attitudes toward mHealth tools, we compared their PU and psychological resistance with other common smartphone applications. Health apps were perceived as moderately useful (Mean = 6.2, SD = 2.7 on a 10-point scale). A Friedman test indicated significant differences in PU across the app categories [χ^2^ (4) = 319.0, *p* < 0.001]. *Post-hoc* pairwise Wilcoxon signed-rank tests with Bonferroni correction revealed that the PU of health apps was significantly lower than that of news apps (Mean = 6.8, SD = 2.6; adjusted *p* < 0.001). However, it was not significantly different from video streaming apps (Mean = 6.4, SD = 2.9; adjusted *p* = 1.000) or social networking social networking apps (Mean = 5.9, SD = 3.1; adjusted *p* = 0.345). Detailed descriptive statistics and score distributions for all app categories are available in Supplementary Figure 1.

Regarding psychological resistance to installing an app, measured on a 5-point scale (1 = “no resistance at all” to 5 = “very strong resistance”), participants reported a mean resistance score of 2.69 (SD = 1.10) for a health app recommended by a physician for health management. This level of resistance was not significantly different from the resistance toward a commercial shopping app offering discount incentives (Mean = 2.66, SD = 1.16; Wilcoxon signed-rank test, *p* = 0.521).

### 3.3 Specific reasons for hesitation in installing health apps

To explore the cognitive barriers underlying this resistance, we compared the specific reasons for hesitation in health apps versus shopping apps (Table 2). The most common concerns across both app types were unrelated to health: “Storage/Data Concerns” (Health: 43.1% vs. Shopping: 48.3%) and “Privacy/Security Concerns” (Health: 42.3% vs. Shopping: 46.0%). Concerns about “Low expected use” (30.7% vs. 45.7%, *p* < 0.001) and “lack of necessity” (23.2% vs. 31.2%, *p* < 0.001) were significantly less common for health apps, while no significant differences were found for concerns about “Perceived hassle and effort” (24.5% vs. 22.9%).

**Table 2:** Reasons for hesitation in installing apps.

Reason for hesitation	Health app (%)	Shopping app (%)	χ^2^ (McNemar)	*p*-value	Sig
Storage/data concerns	43.1	48.3	11.1	0.001	***
Privacy/security concerns	42.3	46.0	5.8	0.016	*
Low expected use	30.7	45.7	49.8	< 0.001	***
Usage fees concerns	25.5	23.0	2.3	0.133	
Perceived hassle and effort	24.5	22.9	0.7	0.396	
Lack of necessity	23.2	31.2	17.1	< 0.001	***
Notification concerns	22.5	32.6	34.3	< 0.001	***
Low perceived usefulness	22.0	28.6	12.4	< 0.001	***
Usage uncertainty	9.2	7.1	3.3	0.068	
Already using similar	8.4	8.4	0	> 0.999	
Negative feedback	5.0	5.2	0	> 0.999	
None of the above	7.1	5.7	2.7	0.1	

McNemar’s test was used to compare paired binary responses for each reason. Multiple responses were allowed. *p < 0.05, **p < 0.01, ***p < 0.001.

### 3.4 Subgroup analyses and interaction effects

To evaluate the perception of health apps across different demographic and socioeconomic groups, we examined descriptive subgroup comparisons based on age, sex, educational background, self-rated economic status, and regular hospital visits (Tables 3, 4). Across these strata, overall subgroup means for PU and psychological resistance were relatively similar, whereas a clear proficiency gradient remained visible in most strata: lower smartphone proficiency was generally associated with lower PU and higher resistance. The largest descriptive gap was observed among participants with regular hospital visits, for whom the overall mean PU was 6.2, compared with 3.6 among those reporting no smartphone proficiency. Additional descriptive summaries of overall outcome differences across each subgroup are provided in Supplementary Tables 7–11.

**Table 3:** Perceived usefulness of health apps by subgroups and smartphone proficiency.

Demographic subgroup	Overall mean (SD)	Not at all	Insufficiently	Moderately	Highly
Age					
18–39	6.3 (2.8)	9.0 (NA)	5.0 (2.3)	6.3 (2.7)	7.0 (3.1)
40–59	6.1 (2.7)	5.6 (3.6)	6.1 (2.4)	6.3 (2.7)	5.8 (3.5)
60+	5.9 (2.7)	4.0 (3.2)	5.7 (2.4)	6.4 (2.6)	6.6 (3.1)
Sex					
Male	6.0 (2.8)	5.1 (3.1)	5.0 (2.3)	6.2 (2.7)	6.5 (3.2)
Female	6.3 (2.7)	4.3 (3.6)	6.3 (2.3)	6.4 (2.7)	6.9 (3.2)
Economic status					
Very tight	5.8 (2.9)	2.0 (1.7)	5.6 (2.5)	5.9 (2.9)	6.8 (3.1)
Tight	6.3 (2.5)	4.3 (3.8)	5.9 (2.1)	6.4 (2.5)	7.0 (2.7)
Somewhat comfortable	6.3 (2.6)	5.4 (3.0)	5.9 (2.5)	6.4 (2.5)	7.3 (2.9)
Comfortable	6.1 (3.4)	9.5 (0.7)	4.6 (2.7)	6.8 (3.2)	5.6 (3.8)
Educational background					
High school or below	5.9 (2.8)	3.8 (3.2)	5.6 (2.5)	6.2 (2.8)	6.5 (3.0)
College/diploma	6.3 (2.9)	5.6 (4.0)	6.5 (2.3)	6.0 (3.0)	6.9 (3.5)
University degree	6.3 (2.6)	5.6 (3.3)	5.6 (2.3)	6.5 (2.5)	6.6 (3.2)
Regular hospital visits					
No visits	6.1 (2.8)	5.9 (3.7)	5.3 (2.5)	6.1 (2.6)	6.9 (3.1)
Regular visits	6.2 (2.7)	3.6 (2.8)	6.1 (2.2)	6.5 (2.7)	6.3 (3.3)

Values are presented as Mean (Standard Deviation). PU ranges from 1 to 10.

**Table 4:** Psychological resistance toward health apps by subgroups and smartphone proficiency.

Demographic subgroup	Overall mean (SD)	Not at all	Insufficiently	Moderately	Highly
Age					
18–39	2.5 (1.1)	1.0 (NA)	3.0 (0.9)	2.6 (1.0)	2.3 (1.3)
40–59	2.8 (1.1)	3.4 (1.5)	3.0 (1.0)	2.7 (1.0)	2.6 (1.2)
60+	2.7 (1.2)	3.5 (1.4)	2.8 (1.2)	2.7 (1.1)	1.9 (0.9)
Sex					
Male	2.7 (1.1)	3.3 (1.5)	3.0 (1.2)	2.6 (1.0)	2.3 (1.2)
Female	2.7 (1.1)	3.4 (1.5)	2.8 (1.0)	2.7 (1.0)	2.2 (1.2)
Economic status					
Very tight	3.0 (1.1)	4.3 (1.6)	3.1 (1.0)	2.9 (1.0)	2.5 (1.2)
Tight	2.7 (1.1)	3.5 (1.4)	2.9 (1.1)	2.7 (1.0)	2.1 (1.1)
Somewhat comfortable	2.5 (1.1)	3.2 (1.0)	2.7 (1.1)	2.4 (1.0)	2.3 (1.2)
Comfortable	2.5 (1.3)	1.0 (0.0)	2.9 (1.0)	2.7 (1.3)	2.4 (1.4)
Educational background					
High school or below	2.8 (1.1)	3.9 (1.0)	3.0 (1.1)	2.6 (1.0)	2.5 (1.3)
College/Diploma	2.7 (1.0)	2.8 (1.8)	2.7 (1.0)	2.7 (0.9)	2.4 (1.0)
University Degree	2.6 (1.1)	2.7 (1.6)	3.0 (1.1)	2.6 (1.0)	2.1 (1.2)
Regular hospital visits					
No visits	2.7 (1.1)	3.0 (1.6)	3.0 (1.1)	2.7 (1.0)	2.2 (1.2)
Regular visits	2.7 (1.1)	3.6 (1.3)	2.9 (1.1)	2.6 (1.0)	2.4 (1.3)

Values are presented as Mean (Standard Deviation). Resistance ranges from 1 to 5.

We then tested formal interaction terms between smartphone proficiency and each subgroup variable. No significant interactions were observed for age, sex, or educational background (all *p* > 0.05), whereas significant interactions were identified for self-rated economic status (PU: *p* = 0.039; resistance: *p* = 0.007) and regular hospital visits (PU: *p* = 0.033). These findings suggest heterogeneity in the magnitude of the proficiency effect, but not a general reversal of its direction across strata. Because the “not at all proficient” category was small in some strata, these descriptive contrasts should be interpreted cautiously.

### 3.5 Smartphone proficiency as the primary predictor

To complement these descriptive subgroup comparisons, we then conducted ordinal logistic regression analyses to identify independent predictors of PU and psychological resistance toward health apps (Table 5).

**Table 5:** Multivariable predictors of perceived usefulness and psychological resistance toward health apps (*N* = 680).

Predictor variables	PU: aOR	Sig.	Resistance: aOR	Sig.
	(95% CI)		(95% CI)	
Self-rated smartphone proficiency				
Insufficiently proficient	1.87 (0.82–4.25)		0.36 (0.16–0.85)	*
Moderately proficient	2.91 (1.28–6.59)	*	0.24 (0.10–0.56)	***
Highly proficient	4.69 (1.90–11.52)	***	0.12 (0.05–0.31)	***
Age	1.00 (0.99–1.01)		1.00 (0.99–1.01)	
Sex (female)	1.47 (1.09–2.00)	*	0.87 (0.64–1.20)	
Educational background				
College/diploma	1.11 (0.74–1.66)		1.08 (0.72–1.62)	
University degree	1.17 (0.86–1.60)		0.93 (0.67–1.29)	
Occupational status				
Self-employed	0.73 (0.43–1.24)		0.82 (0.47–1.46)	
Part-time/student	1.29 (0.88–1.90)		0.84 (0.56–1.25)	
Unemployed/homemaker	0.84 (0.59–1.22)		1.11 (0.76–1.63)	
Economic status				
Tight	1.12 (0.78–1.62)		0.72 (0.50–1.05)	
Somewhat comfortable	1.15 (0.77–1.72)		0.53 (0.35–0.79)	**
Comfortable	0.94 (0.48–1.84)		0.71 (0.36–1.41)	
Hospital (regular visits)	1.10 (0.82–1.48)		0.89 (0.65–1.21)	
Exercise habit				
Irregularly (1–2 days/month)	2.18 (1.41–3.39)	***	0.62 (0.39–0.99)	*
Regularly (1–2 days/week)	2.01 (1.38–2.94)	***	0.92 (0.63–1.36)	
Regularly (≥3 days/week)	2.73 (1.93–3.88)	***	0.62 (0.44–0.89)	**
Self-rated health status	0.99 (0.98–1.00)		1.00 (0.99–1.01)	
WHO-5 wellbeing index	1.03 (1.00–1.06)	*	0.99 (0.96–1.02)	

aOR, Adjusted Odds Ratio; CI, Confidence Interval. *p < 0.05, **p < 0.01, ***p < 0.001.

Reference categories: smartphone proficiency = Not at all proficient; sex = male; educational background = high school or below; occupational status = employed; economic status = very tight; regular hospital visits = no visits; exercise habit = almost none.

The most consistent predictors for both outcomes were self-rated smartphone proficiency and exercise habits. Compared to those with no proficiency, participants with moderate and high proficiency demonstrated significantly higher PU (adjusted OR [aOR] = 2.91 and aOR = 4.69, respectively) and markedly lower psychological resistance (aOR = 0.24 and 0.12, respectively). Similarly, a regular exercise habit (≥3 days/week) was a robust predictor of both higher PU (aOR = 2.73, *p* < 0.001) and lower resistance (aOR = 0.62, *p* < 0.01) compared to having almost no exercise habit.

Specific sociodemographic and health-related factors showed outcome-specific associations. Being female was associated only with higher PU (aOR = 1.47, *p* < 0.05), with no significant association with resistance. Self-rated health status was not significantly associated with PU, whereas the WHO-5 reached statistical significance, although its effect size was small (aOR = 1.03). For psychological resistance, self-rated economic status also showed a partial association, with participants reporting somewhat comfortable economic status exhibiting lower resistance than those reporting very tight economic circumstances. In contrast, regular hospital visits, age, educational background, and occupational status were not significantly associated with either PU or resistance.

## 4 Discussion

### 4.1 Principal findings

Our study shows that users’ perceptions toward health apps are not exceptional. Despite their potential to support personal health management, health apps were perceived as only moderately useful, comparable to social media, but inferior to news apps. Participants also displayed a moderate level of psychological resistance towards physician-recommended health apps, and this resistance was indistinguishable from their reluctance to install a commercial shopping app. Rather than a lack of perceived necessity, practical and mundane barriers such as device storage and privacy concerns appeared to drive this resistance. While some health-related indicators showed small associations with PU, smartphone proficiency emerged as the most consistent predictor of both PU and psychological resistance. These findings suggest that, at the initial adoption stage, even physician-recommended health apps are evaluated through the same practical lens as ordinary commercial apps.

### 4.2 The illusion of health needs and practical barriers

Our results indicate that health apps are not perceived as a uniquely valuable category; their PU was comparable to social networking apps and lower than news apps. This dynamic is possibly explained by different value propositions. While social media and news apps provide instant hedonic value fulfilling users’ immediate wants and needs (34–37), the benefits of health apps are often delayed and less tangible. Furthermore, health apps require sustained effort, such as manual data entry (18, 22, 23, 38, 39), which can act as a significant barrier.

This contrast highlights the discrepancy between the potential long-term health benefits and how users perceive usefulness. It suggests that the absence of an immediate hedonic appeal can outweigh performance expectations (22, 40), consistent with extended technology acceptance models that emphasize affective and hedonic factors in mHealth use (9, 41). From a behavioral economics perspective, this discrepancy may also be amplified by temporal discounting. This occurs when delayed, nonmonetary rewards are undervalued compared to immediate costs (42).

A paradox therefore emerged regarding psychological resistance. Although participants cited a “lack of necessity” significantly less often for health apps than shopping apps, their overall resistance to installing them was virtually identical. This suggests that, even when users cognitively recognize the necessity of a health app, this motivation may be insufficient to overcome the immediate “costs” of adoption, such as limited device storage, the cognitive burden of navigating an unfamiliar interface, and data privacy anxieties (43, 44).

Consequently, these practical barriers may function as absolute blockers rather than mere inconveniences. This finding challenges the prevailing assumption that health-related needs inherently drive mHealth adoption.

### 4.3 Smartphone proficiency and digital inequality

Unexpectedly, smartphone proficiency outweighs actual health needs in predicting user attitudes toward health apps (24, 45). For less proficient users, digital unfamiliarity may diminish PU and heighten resistance, regardless of any app design issues. In this sense, the initial hassle of installation may overshadow the long-term clinical benefits. Importantly, subgroup analyses suggest this pattern is not confined to a single demographic segment. The direction of the proficiency gradient is preserved across age, sex, and educational strata, though its magnitude varies with self-rated economic status and regular hospital visits. These findings suggest that smartphone proficiency is a relevant prerequisite that interacts with social and clinical vulnerability to shape the strength of resistance.

These findings highlight a critical dimension of the digital divide in health, moving beyond the traditional focus on physical access (16, 46). Although much of the current literature emphasizes third-level disparities in the utilization of digital outcomes (46), this does not imply that foundational skill gaps have closed. Rather, our findings demonstrate that the “second-level digital divide” remains a significant obstacle to the initial adoption of mHealth (47, 48).

Building on the concept of active opposition, our findings reveal a concerning clinical dynamic. Individuals with lower smartphone proficiency demonstrated significantly stronger psychological resistance even when health apps were recommended by healthcare professionals. This response may stem from a deeper resistance rooted in a perceived incongruence with one’s abilities (14). This pronounced frustration aligns with “technostress”—psychological stress triggered by the mandated use of new technology (49–51). Consequently, a physician’s well-intentioned recommendation may prove counterproductive if perceived as an overwhelming obligation. Such recommendations can trigger a cascade of negative psychological responses, including feelings of inadequacy and active psychological reactance (52). The consequences may extend beyond simple non-use, potentially affecting patients’ engagement with digital health tools and their willingness to participate in proactive health management (53).

Given these dynamics, digital skill support is not an optional adjunct but a fundamental prerequisite for ethical and effective health app implementation (54, 55). Rather than focusing only on designing “better apps,” researchers and practitioners should also address foundational digital capability development (56, 57), which is increasingly recognized as a social determinant of health (58, 59). In the digital era, establishing a baseline of digital competency is a central component of any meaningful public health intervention (60, 61).

### 4.4 Limitations and future research directions

Several limitations of this study should be acknowledged. First, the cross-sectional design precludes causal inferences between smartphone proficiency, PU, and psychological resistance.

Second, recruiting via an online internet panel introduces selection bias by overrepresenting individuals with baseline digital literacy. Accordingly, our study likely overestimates PU and underestimates psychological resistance relative to the general Japanese adult population, implying that the real-world digital divide may be even more severe than our data suggest. Generalizability to populations with lower digital access, non-internet users, or different cultural contexts may therefore be limited (62).

Third, comparing health apps with commercial categories (e.g., news, gaming) introduces a potential framing bias. This relative measurement may have systematically disadvantaged respondents who do not actively use or value those comparator apps, potentially skewing their PU scores.

Finally, reliance on non-validated, single-item measures of PU and resistance developed by the authors limits the psychometric robustness and comparability of the study. Furthermore, this study focuses solely on self-reported attitudes and does not consider objective behavior. Future studies should incorporate standardized scales and objective paradata approaches (i.e., in-app usage metrics or system logs as an objective complement to self-reports) (63, 64) to further validate the skill-dependent adoption pathways.

## 5 Conclusion

Health apps are not perceived as uniquely valuable. They face limits in perceived usefulness and practical installation barriers comparable to those of everyday commercial apps. Notably, users’ basic smartphone proficiency was a more consistent predictor of perceived usefulness and resistance to adoption than health-related indicators, even when apps were recommended by physicians. To ensure equitable mHealth interventions, public health strategies must prioritize improving users’ basic digital skills.

## Data Availability

The datasets presented in this article are not readily available because approval by the Institutional Review Board is required for data sharing to ensure participant privacy and ethical compliance. Requests to access the datasets should be directed to inagaki@ds.nagoya-cu.ac.jp.
