# Supplementary material for: Why health apps fail: the role of smartphone proficiency in mHealth resistance
Source: Front Public Health. 2026 May 26;14:1829773. doi: 10.3389/fpubh.2026.1829773 (PMC13246712; doi:10.3389/fpubh.2026.1829773)
Supplement: Supplementary file 1 [file Supplementary_file_1.pdf]

Supplementary Materials

1 Supplementary Materials

Supplementary Table 1: Cross-tabulation of Age Group and Daily Smartphone Usage Time
Supplementary Table 2: Self-Reported Smartphone Proficiency by Age Group
Supplementary Table 3: Health App Usage and Features
Supplementary Table 4: Distribution of Health App Usage Status Across Age Groups
Supplementary Table 5: Duration of Use and Reasons for Discontinuation among Former Health App Users (n=118)
Supplementary Table 6: Types of Discontinued Health Apps and Discontinuation Rates (n = 377)
Supplementary Table 7. Descriptive Outcomes and Participant Characteristics by Age Group
Supplementary Table 8. Descriptive Outcomes and Participant Characteristics by Sex
Supplementary Table 9. Descriptive Outcomes and Participant Characteristics by Educational Background
Supplementary Table 10. Descriptive Outcomes and Participant Characteristics by Self-Rated Economic Status
Supplementary Table 11. Descriptive Outcomes and Participant Characteristics by Regular Hospital Visits
Supplementary Figure 1: Distributions and Descriptive Statistics of Perceived Usefulness Across App Types

2 Supplementary Figures and Tables

2.1 Supplementary Table 1: Cross-tabulation of Age Group and Daily Smartphone Usage Time

Age Group	Less than 1 hour		1-2 hours		2-3 hours		3-4 hours		4-6 hours		6+ hours		Total	
**18-39**	2.4%	(6)	6.7%	(17)	8.7%	(22)	20.9%	(53)	27.6%	(70)	33.9%	(86)	100%	(254)
**40-59**	8.1%	(20)	18.7%	(46)	16.3%	(40)	23.2%	(57)	18.3%	(45)	15.4%	(38)	100%	(246)
**60+**	27.6%	(60)	24.0%	(52)	18.4%	(40)	18.0%	(39)	5.1%	(11)	6.9%	(15)	100%	(217)
**Total**	12.0%	(86)	16.0%	(115)	14.2%	(102)	20.8%	(149)	17.6%	(126)	19.4%	(139)	100%	(717)

2.2 Supplementary Table 2: Self-Reported Smartphone Proficiency by Age Group

Age Group	Not at all proficient		Insufficiently proficient		Moderately proficient		Highly proficient		Total	
**18-39**	0.4%	(1)	11.8%	(30)	59.8%	(152)	28.0%	(71)	100%	(254)
**40-59**	2.8%	(7)	35.0%	(86)	52.0%	(128)	10.2%	(25)	100%	(246)
**60+**	7.8%	(17)	38.2%	(83)	45.6%	(99)	8.3%	(18)	100%	(217)
**Total**	3.5%	(25)	27.8%	(199)	52.9%	(379)	15.9%	(114)	100%	(717)

2.3 Supplementary Table 3: Health App Usage and Features

Variable	Category	n	(%)
Health App Usage	Never used	320	(44.6)
	Used to use	118	(16.5)
	Currently use	279	(38.9)
Health App Usage Frequency (Currently Using, n=279)	0 days/week	16	(5.7)
	1 day/week	27	(9.7)
	2 days/week	20	(7.2)
	3 days/week	13	(4.7)
	4 days/week	5	(1.8)
	5 days/week	18	(6.5)
	6 days/week	6	(2.2)
	7 days/week (Daily)	174	(62.4)
Health App Usage Duration (Currently Using, n=279)	< 1 month	14	(5.0)
	1-12 months	61	(21.9)
	1-3 years	81	(29.0)
	3+ years	105	(37.6)
	Don't remember	18	(6.5)
Tracked Items (Currently Using, n=279)	Exercise tracking	192	(68.8)
	Weight management	121	(43.4)
	Sleep tracking	71	(25.4)
	Period tracking	52	(18.6)
	Blood pressure tracking	37	(13.3)
	Diet management	31	(11.1)
	General health tracking	29	(10.4)
	Medication tracking	16	(5.7)
	Blood glucose tracking	6	(2.2)
	Skin condition tracking	5	(1.8)

Note: For 'Tracked Items', participants could select multiple options. Percentages are based on the number of current health app users (n=279).

2.4 Supplementary Table 4: Distribution of Health App Usage Status Across Age Groups

Age Group	Currently Using [% (n)]		Used in the Past [% (n)]		Never Used [% (n)]		Total [% (n)]	
**18-39**	42.5%	(108)	19.7%	(50)	37.8%	(96)	100%	(254)
**40-59**	37.0%	(91)	16.7%	(41)	46.3%	(114)	100%	(246)
**60+**	36.9%	(80)	12.4%	(27)	50.7%	(110)	100%	(217)
**Total**	38.9%	(279)	16.5%	(118)	44.6%	(320)	100%	(717)

Note: "Currently Using" refers to participants currently using at least one health app. "Used in the Past" refers to participants who previously used health apps but are no longer using them. "Never Used" refers to participants who have never used any health app. Percentages are row-based and calculated based on the total number of participants in each age group. Due to rounding, percentages may not always sum to exactly 100%.

2.5 Supplementary Table 5: Duration of Use and Reasons for Discontinuation among Former Health App Users (n=118)

Variable	Category	n	(%)
Duration of Use	A few days	2	1.7
(Used to Use, n=118)	Less than 1 month	7	5.9
	1-3 months	24	20.3
	3-6 months	14	11.9
	6-12 months	24	20.3
	1-3 years	13	11.0
	More than 3 years	8	6.8
	Don't remember	26	22.0
Reason for Discontinuation	Poor usability	23	19.5
(Used to Use, n=118)	Lost interest	22	18.6
	No specific reason	22	18.6
	Forgetting to track	20	16.9
	Perceived low effectiveness	18	15.3
	Can manage health without the app	17	14.4
	Too much effort (e.g., data entry)	14	11.9
	Other	12	10.2
	Lack of time	9	7.6
	Achieved app's purpose	8	6.8
	Stopped managing health/exercise	8	6.8
	Fees/Costs	7	5.9
	App-related issues (service changes)	3	2.5

Note: For 'Reason for Discontinuation', participants could select multiple reasons. Percentages are calculated based on the number of former health app users (n=118).

2.6 Supplementary Table 6: Types of Discontinued Health Apps and Discontinuation Rates (n = 377)

Variable	Category	Lifetime Users (A)	Discontinued Users (B)	Discontinuation Rate (%) [(B/A) × 100]
Tracked Items	Weight management	186	65	34.9%
	Exercise tracking	260	68	26.2%
	Diet management	71	40	56.3%
	Sleep tracking	120	49	40.8%
	Medication tracking	26	10	38.5%
	Period tracking	77	25	32.5%
	Blood pressure tracking	47	10	21.3%
	Blood glucose tracking	10	4	40.0%
	Skin condition tracking	6	1	16.7%
	General health tracking	41	12	29.3%

Note: (A) "Lifetime Users" refers to the total number of participants who reported having used a specific health app feature. (B) "Discontinued Users" refers to the number of participants who were lifetime users (A) but are not currently using the feature. The "Discontinuation Rate (%)" was calculated as [(B) Discontinued Users / (A) Lifetime Users] × 100.
Among the 397 participants who reported current or past use of health apps, 20 individuals did not specify the features they used and were thus excluded from this feature-specific analysis. Consequently, the data in this table were extracted from the remaining 377 unique individuals who reported having used at least one health app feature.

2.7 Supplementary Table 7: Descriptive Outcomes and Participant Characteristics by Age Group

Characteristic	18-39<br>N = 254[1]	40-59<br>N = 246[1]	60+<br>N = 217[1]
**Perceived Usefulness of Health App**	6.3 (2.8)	6.1 (2.7)	5.9 (2.7)
**Perceived Resistance toward Health App**	2.5 (1.1)	2.8 (1.1)	2.7 (1.2)
**Self-rated health status (0–100)**	64.2 (23.2)	67.2 (21.5)	68.1 (20.2)
**WHO-5 Well-being Index (0-25)**	12.3 (6.1)	11.9 (6.2)	14.3 (5.5)
**Smartphone Proficiency**			
Not at all	1 (0.4%)	7 (2.8%)	17 (7.8%)
Insufficiently proficient	30 (12%)	86 (35%)	83 (38%)
Moderately proficient	152 (60%)	128 (52%)	99 (46%)
Highly proficient	71 (28%)	25 (10%)	18 (8.3%)
**Health App Usage Status**			
Never used	96 (38%)	114 (46%)	110 (51%)
Used to use	50 (20%)	41 (17%)	27 (12%)
Currently use	108 (43%)	91 (37%)	80 (37%)

Note:
Perceived Usefulness was assessed on a 10-point scale (1 = strongly disagree, 10 = strongly agree). Perceived Resistance was assessed on a 5-point scale (1 = no resistance at all, 5 = very strong resistance).

[1] Mean (SD); n (%)

2.8 Supplementary Table 8: Descriptive Outcomes and Participant Characteristics by Sex

Characteristic	Male<br>N = 348[1]	Female<br>N = 369[1]
**Perceived Usefulness of Health App**	6.0 (2.8)	6.3 (2.7)
**Perceived Resistance toward Health App**	2.7 (1.1)	2.7 (1.1)
**Self-rated health status (0–100)**	66.6 (22.2)	66.3 (21.3)
**WHO-5 Well-being Index (0-25)**	13.2 (6.4)	12.4 (5.7)
**Smartphone Proficiency**		
Not at all	11 (3.2%)	14 (3.8%)
Insufficiently proficient	83 (24%)	116 (31%)
Moderately proficient	182 (52%)	197 (53%)
Highly proficient	72 (21%)	42 (11%)
**Health App Usage Status**		
Never used	153 (44%)	167 (45%)
Used to use	61 (18%)	57 (15%)
Currently use	134 (39%)	145 (39%)

Note:
Perceived Usefulness was assessed on a 10-point scale (1 = strongly disagree, 10 = strongly agree). Perceived Resistance was assessed on a 5-point scale (1 = no resistance at all, 5 = very strong resistance).

[1] Mean (SD); n (%)

2.9 Supplementary Table 9: Descriptive Outcomes and Participant Characteristics by Educational Background

Characteristic	High school or below<br>N = 237[1]	College/Diploma<br>N = 125[1]	University Degree<br>N = 355[1]
**Perceived Usefulness of Health App**	5.9 (2.8)	6.3 (2.9)	6.3 (2.6)
**Perceived Resistance toward Health App**	2.8 (1.1)	2.7 (1.0)	2.6 (1.1)
**Self-rated health status (0–100)**	66.2 (22.2)	66.4 (23.9)	66.6 (20.7)
**WHO-5 Well-being Index (0-25)**	12.4 (6.0)	12.9 (6.2)	12.9 (6.0)
**Smartphone Proficiency**			
Not at all	13 (5.5%)	5 (4.0%)	7 (2.0%)
Insufficiently proficient	61 (26%)	38 (30%)	100 (28%)
Moderately proficient	130 (55%)	64 (51%)	185 (52%)
Highly proficient	33 (14%)	18 (14%)	63 (18%)
**Health App Usage Status**			
Never used	111 (47%)	54 (43%)	155 (44%)
Used to use	35 (15%)	22 (18%)	61 (17%)
Currently use	91 (38%)	49 (39%)	139 (39%)

Note:
Perceived Usefulness was assessed on a 10-point scale (1 = strongly disagree, 10 = strongly agree). Perceived Resistance was assessed on a 5-point scale (1 = no resistance at all, 5 = very strong resistance).

[1] Mean (SD); n (%)

2.10 Supplementary Table 10: Descriptive Outcomes and Participant Characteristics by Self-Rated Economic Status

Characteristic	Very tight<br>N = 149[1]	Tight<br>N = 272[1]	Somewhat comfortable<br>N = 212[1]	Comfortable<br>N = 47[1]
**Perceived Usefulness of Health App**	5.8 (2.9)	6.3 (2.5)	6.3 (2.6)	6.1 (3.4)
**Perceived Resistance toward Health App**	3.0 (1.1)	2.7 (1.1)	2.5 (1.1)	2.5 (1.3)
**Self-rated health status (0–100)**	54.4 (23.7)	66.6 (19.9)	71.9 (20.1)	80.6 (15.4)
**WHO-5 Well-being Index (0-25)**	9.1 (5.5)	12.7 (5.7)	14.7 (5.5)	17.7 (5.1)
**Smartphone Proficiency**				
Not at all	6 (4.0%)	6 (2.2%)	11 (5.2%)	2 (4.3%)
Insufficiently proficient	52 (35%)	69 (25%)	59 (28%)	8 (17%)
Moderately proficient	70 (47%)	163 (60%)	107 (50%)	20 (43%)
Highly proficient	21 (14%)	34 (13%)	35 (17%)	17 (36%)
**Health App Usage Status**				
Never used	74 (50%)	114 (42%)	93 (44%)	21 (45%)
Used to use	25 (17%)	47 (17%)	33 (16%)	8 (17%)
Currently use	50 (34%)	111 (41%)	86 (41%)	18 (38%)

Note:
Perceived Usefulness was assessed on a 10-point scale (1 = strongly disagree, 10 = strongly agree). Perceived Resistance was assessed on a 5-point scale (1 = no resistance at all, 5 = very strong resistance).

[1] Mean (SD); n (%)

2.11 Supplementary Table 11: Descriptive Outcomes and Participant Characteristics by Regular Hospital Visits

Characteristic	No visits N = 372[1]	Regular visits N = 345[1]
**Perceived Usefulness of Health App**	6.1 (2.8)	6.2 (2.7)
**Perceived Resistance toward Health App**	2.7 (1.1)	2.7 (1.1)
**Self-rated health status (0–100)**	71.7 (19.2)	60.8 (22.9)
**WHO-5 Well-being Index (0-25)**	13.5 (6.0)	11.9 (6.0)
**Smartphone Proficiency**		
Not at all	11 (3.0%)	14 (4.1%)
Insufficiently proficient	90 (24%)	109 (32%)
Moderately proficient	199 (53%)	180 (52%)
Highly proficient	72 (19%)	42 (12%)
**Health App Usage Status**		
Never used	174 (47%)	146 (42%)
Used to use	62 (17%)	56 (16%)
Currently use	136 (37%)	143 (41%)

Note:
Perceived Usefulness was assessed on a 10-point scale (1 = strongly disagree, 10 = strongly agree). Perceived Resistance was assessed on a 5-point scale (1 = no resistance at all, 5 = very strong resistance).

[1] Mean (SD); n (%)

2.12 Supplementary Figure 1

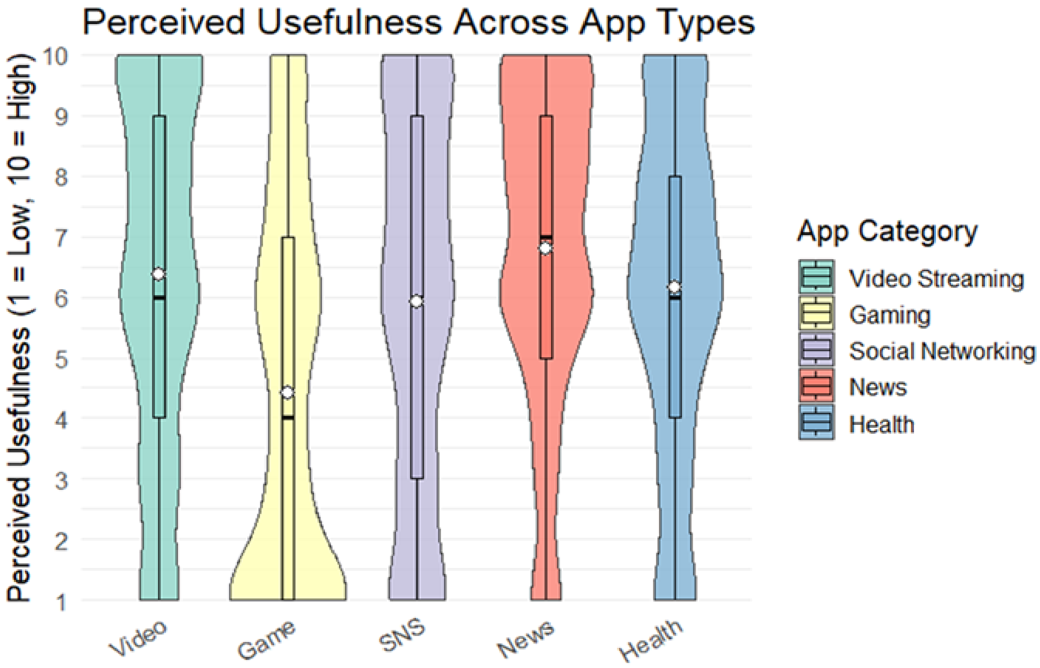

Descriptive Statistics of Perceived Usefulness by App Type

	Video	Game	SNS	News	Health
Mean	6.4	4.4	5.9	6.8	6.2
Median	6	4	6	7	6
SD	2.9	3.1	3.1	2.6	2.7

Supplementary Figure 1: Distributions and Descriptive Statistics of Perceived Usefulness Across App Types

Note. Perceived Usefulness was assessed on a 10-point Numerical Rating Scale (NRS; 1 = strongly disagree, 10 = strongly agree; see Methods for full item details). Note that the y-axis labels "Low" and "High" correspond to "strongly disagree" and "strongly agree", respectively. Violin plots illustrate score distributions; nested box plots show medians and interquartile ranges (outliers are not displayed). White points indicate mean scores. The table below lists key descriptive statistics. X-axis abbreviations: Video (Video Streaming), Game (Gaming), SNS (Social Networking), News (News Apps), Health (Health Apps).
A Friedman test indicated significant differences across the five app categories ($\chi^2(4) = 319.0$, $p < .001$). Post-hoc pairwise Wilcoxon signed-rank tests with Bonferroni correction revealed that the perceived usefulness of Health apps was significantly different from Gaming apps (adjusted $p < .001$) and News apps (adjusted $p < .001$). No significant differences were observed between Health apps and Video Streaming apps (adjusted $p = 1.000$), or Social Networking Services (adjusted $p = .345$).
